# Effects of bone morphogenic protein 4 (BMP4) and its inhibitor, Noggin, on *in vitro *maturation and culture of bovine preimplantation embryos

**DOI:** 10.1186/1477-7827-9-18

**Published:** 2011-02-01

**Authors:** Isabel La Rosa, Luiz SA Camargo, Michele M Pereira, Rafael Fernandez-Martin, Dante A Paz, Daniel F Salamone

**Affiliations:** 1 Laboratory of Animal Biotechnology, Agriculture Faculty, University of Buenos Aires (UBA), Argentina; 2 Embrapa Dairy Cattle, Reproduction and Biotechnology, Dom Bosco, Juiz de Fora, M. G., Brazil; 3 Laboratory of Developmental Biology BBED, Exact and Natural Sciences Faculty, UBA, IFIBYNE-CONICET, Argentina; 4 National Institute of Scientific and Technological Research (CONICET), Argentina

## Abstract

**Background:**

BMP4 is a member of the transforming growth factor beta (TGFbeta) superfamily and Noggin is a potent BMP inhibitor that exerts its function by binding to BMPs preventing interactions with its receptors. The aim of this work was to investigate the role of BMP4 and Noggin, on oocytes *in vitro *maturation (m experiments) and embryos *in vitro *development (c experiments) of bovine.

**Methods:**

For m experiments, COCs were collected from slaughterhouse ovaries and *in vitro *matured in TCM with 100 ng/ml of either BMP4 or Noggin. After 24 h, the nuclear stage of the oocytes was determined by staining with Hoechst 33342. In addition, RT-qPCR was performed on MII oocytes to study the relative concentration of *ZAR1, GDF9, BAX, MATER *and *HSP70 *transcripts. Treated oocytes were submitted to parthenogenic activation (PA) or *in vitro *fertilization (IVF) and cultured in CR2. For c experiments, non-treated matured oocytes were submitted to PA or IVF to generate embryos that were exposed to 100 ng/ml of BMP4 or Noggin in CR2 until day nine of culture. Cleavage, blastocyst and hatching rates, expression pattern of the transcription factor Oct-4 in blastocysts and embryo cell number at day two and nine post-activation or fertilization were evaluated.

**Results:**

We found that Noggin, as BMP4, did not affect oocyte nuclear maturation. Noggin supplementation up-regulated the expression of *HSP70 and MATER *genes in matured oocytes. Moreover, BMP4 during maturation increased the proportion of Oct-4 positive cells in parthenogenic embryos. On the other hand, when Noggin was added to embryo culture medium, developmental rates of parthenogenic and *in vitro *fertilized embryos were reduced. However, BMP4 addition decreases the development only for *in vitro *fertilized embryos. BMP4 and Noggin during culture reduced the proportion of Oct-4-expressing cells.

**Conclusions:**

Our results show that BMP4 is implicated in bovine oocytes maturation and embryo development. Moreover, our findings demonstrate, for the first time, that a correct balance of BMP signaling is needed for proper pre-implantation development of bovine embryos.

## Background

Bone Morphogenetic Protein 4 is a member of the transforming growth factor beta (TGFβ) superfamily which controls numerous events of embryonic, fetal and even adult development in all vertebrates [1]. Intracellular mediators of BMPs are Smad proteins which form a complex that is then translocated to the nucleus and regulates gene expression [2]. Noggin is a potent inhibitor of BMPs that directly binds to BMP members, specifically 2, 4 and 7, and blocks the sites required for interaction with BMP receptors. It belongs to the so called "ligand traps" group [3] and is a critical regulator of BMP signaling.

BMP4 is expressed first in stromal cells and later in follicular development, in theca cells. Its receptors are found in granulosa cells as well as in the oocyte itself [4,5] and it is in charge of primordial to primary follicular transition, stimulates granulose cells proliferation, pre-antral follicular growth and follicular survival and regulates steroideogenesis in granulosa cells [6]. Other TGFβ members, such as BMP15 and GDF9, are expressed by the oocyte also in many species as cats [7], mice [8], rats [9], hens [10], goats [11], and cattle [12]. The importance of BMPs for the female reproductive system has been evidenced by spontaneous mutations of these genes in sheep [13,14] influencing ovulation rates. Of all the ovarian BMPs the most associated with the stem cells properties is in fact BMP4. Therefore, it could be a potencial regulator of this cell capacity in bovine embryos. BMP4 maintains the pluripotency of primordial germ cells [15] and embryonic stem cells (ESC) in mice [16]. In contrast, BMP4 inhibition with Noggin is required to maintain pluripotency of embryonic stem cells in the human [17]. In domestic species, ESCs have not been established yet but a study by Pant and Keefer [18] showed that BMP4 inhibition with Noggin increases the amount of Nanog transcripts (a pluripotent-related factor) in bovine inner cell mass (ICM) explants. To date, the specific role of this factor in bovine pluripotent state has not been elucidated. The POU transcription factor Oct-4, which is also related to pluripotency, is not expressed only in the ICM in bovine but it could be implicated in trophoblastic expansion where intense cellular division precedes differentiation and so it is important for embryo implantation [19,20]. Moreover, in later developmental stages, BMP4 induces embryonic and extraembryonic mesoderm formation [21,22] and is also related to vasculogenesis [23] in the embryo as well as in the developing placenta. As a result it is of major importance in the success of pregnancy.

The aim of this work was to investigate the role of BMP4 and its inhibitor, Noggin, in oocyte maturation and development of bovine embryos produced by parthenogenesis and *in vitro *fertilization. Several developmental and molecular aspects were evaluated. These included meiotic stage and the amount of several transcripts in oocytes, cleavage and blastocyst rates, embryo cell number and Oct-4 protein expression.

## Methods

### Reagents

Except where otherwise indicated, all chemicals were obtained from Sigma Chemical Company (St. Louis, MO, USA).

### Experimental design

#### Maturation (m) experiments

Oocytes were *in vitro *matured under three different conditions [BMP4 (mBMP4), Noggin (mNoggin) and Control (mControl)] and then submitted to parthenogenic activation (PA) or *in vitro *fertilization (IVF). Nuclear stage was analyzed and transcripts of different genes were quantified by RT-qPCR on oocytes matured under the three treatments.

#### Culture (c) experiments

Embryos were produced by PA or IVF and then cultured in CR2 medium (cControl) or with the addition of BMP4 (cBMP4) or Noggin (cNoggin).

For both m and c experiments, numbers of cleaved embryos and their cell numbers were recorded on day two and numbers of blastocysts and their cell numbers were recorded on day nine post fertilization/activation. Oct-4 immunostaining was performed on blastocysts from the three treatments.

### Oocyte collection and maturation

Bovine ovaries were obtained from a slaughter house and maintained at 25°C until processed. Cumulus-oocyte complexes (COCs) were aspirated using an 18-G needle and a 10 ml syringe and rinsed in phosphate buffered solution (PBS, Gibco 14190, Grand Island, NY, USA) supplemented with 1% antibiotic-antimycotic (Gibco 15240), and 10% Adult Bovine Serum (Internegocios, Mercedes, Buenos Aires, Argentine). Maturation was performed in 100 μl microdroplets of medium 199 (TCM, Gibco 11150) supplemented with 10% Fetal Bovine Serum (FBS, Internegocios), 1% antibiotic-antimycotic, 20 μM cysteamine (M-9768), 0.1 mM sodium pyruvate (S-8636), and 2 mM FSH (Folltropin-V, Bioniche, Belleville, ON, Canada), under mineral oil (M-8410). Maturation conditions were 6.5% CO_2 _in humidified atmosphere at 39°C.

For m experiments, COCs were matured as described above but FBS was replaced by 0.6% w/v fatty acid-free bovine serum albumin (faf BSA, A-6003). COCs were matured in the presence of 100 ng/ml of BMP4 (314-bp-010, R&D Systems, Minneapolis, MN, USA) or Noggin (N-6784). A Control group consisted of COCs matured without BMP4 or Noggin.

### Nuclear maturation

Cumulus cells were removed from COCs by vortexing for 3 min in 1mg/ml hyaluronidase (H-4272) in Hepes-buffered Tyrode's medium containing albumin, lactate and pyruvate (TALP-H). After several rinses in TALP-H, DNA was stained with 2 μg/ml of Hoechst 33342 (B-2261) in TCM for 10 min and oocyte nuclear status was determined under UV light on an inverted microscope. A holding pipette was used to rotate the oocytes.

### Parthenogenic activation

Denuded oocytes were chemically activated in TALP-H with 5μM ionomycin (I24222, Invitrogen, Chicago, IL, USA) for 4 min, rinsed several times in TALP-H and immediately incubated in a 100 μl microdrop of TCM containing 1.9 mM 6-diaminopuridine (D-2629), for 3 h. Afterwards, oocytes were rinsed four times in TALP-H and transferred to culture medium.

### *In vitro *fertilization

Frozen bovine sperm were thawed at 35°C in a water-bath and centrifuged twice at 490 g in BO solution [24] supplemented with 0.4% caffeine (C-4144) and 0.02% heparin (H-3149) (Sperm washing solution, SWS). The pellet was resuspended in 50% SWS and 50% BO solution supplemented with 2% faf BSA. Sperm concentrations were adjusted to 20 × 10^6 ^/ml and 100 μl-microdrops were placed under mineral oil. Following 22 h maturation, COCs were rinsed in TALP-H and co-incubated with sperm for 5 h. Presumptive zygotes were vortexed for 30 sec and rinsed three times in TALP-H before being transferred to culture medium.

### Embryo culture

For m experiments, presumptive zygotes were cultured in 100 μl microdrops of serum-free CR2, without co-culture. For c experiments, they were randomly assigned to one of the treatments according to the experimental design (i.e. serum-free CR2 without co-culture (Control) or supplemented with 100 ng/ml of either BMP4 or Noggin). Culture conditions were 6.5% CO_2 _in humidified atmosphere at 39°C. Fifty percent of the culture medium was renewed on days 2, 5 and 7 of culture.

### Embryo development

Cleavage, blastocyst and hatching rates were assessed on day 2 and 9 post fertilization/activation. On day 2, embryonic cell numbers were recorded using an inverted microscope.

Blastocyst total cell numbers were assessed on day 9 of culture, by nuclear staining with 2 μg/ml of Hoechst 33342 in CR2 for 10 min. Then, embryos were placed between a slide and a coverslip and nuclei were counted under UV light using an inverted microscope.

### RNA extraction, quantitative Real Time-Polymerase Chain Reaction (RT-qPCR)

Denuded oocytes were classified by the presence of the first polar body, and kept in RNA later ^® ^(AM 7020, Ambion, Foster City, CA, USA) at -20°C until RNA extraction. Total RNA extraction was performed using a Rneasy Micro kit (Qiagen, Valencia, CA, USA) protocol, according to the manufacturer's instructions, and treated with DNAse. Reverse transcriptions were performed with total RNA from 3 pools of 10 oocytes, using a Superscript™ III first strand synthesis kit (Invitrogen). Relative quantification was performed in triplicate using real time PCR (ABI Prism1 7000, Applied Biosystems, Foster City, CA, USA) and reactions using a mixture of iTaqTM SYBR1Green Supermix with ROX (Bio-Rad, Waltham, MA, USA) with cDNA equivalent to 1.2 oocytes and gene specific primers. Template cDNA was denatured at 95°C for 10 min, followed by 45 cycles of 95°C for 15 sec; gene-specific primer annealing temperature was applied for 30 sec, and elongation was carried out at 72°C for 45 sec/60°C for 30 min (Table 1). After each PCR run, a melting curve analysis was performed for each sample to confirm that a single specific product was generated. Amplicon size was confirmed by ethidium bromide stained-2% agarose gel electrophoresis. Negative Controls, comprised of the PCR reaction mix without nucleic acid, were also run with each group of samples.

**Table 1:** Primer sequences and PCR conditions

Gene	Primer Sequence	Annealing Temperature	Product Length (bp)	GenBank n° of access/Reference
***HSP70***	F 5'AACAAGATCACCATCACCAACG3' R 5'TCCTTCTCCGCCAAGGTGTTG3'	59°C	275	NM174550
***BAX***	F 5'TTTGCTTCAGGGTTTCATCCAGGA3' R 5'CAGCTGCGATCATCCTCTGCAG3'	64°C	174	NM173894
***ZAR1***	F 5'TGCCGAACATGCCAGAAG3' R 5'TCACAGGATAGGCGTTTGC3'	53°C	188	NM_001076203
***MATER***	F 5'TAATGACGACGCTGTGTTCTG3' R 5'GCGGTTCTCAGGTTCTTCAG3'	53°C	206	NM_001007814
***GDF9***	F 5'GACCCCTAAATCCAACAGAA3' R 5'AGCAGATCCACTGATGGAA3'	53°C	120	NM_174681
***β*-*ACTIN *(endogenous)**	F 5'GACATCCGCAAGGACCTCTA3' R 5'ACATCTGCTGGAAGGTGGAC3'	53°C	205	NM_173979
***GAPDH *(endogenous)**	F 5'CCAACGTGTCTGTTGTGGATCTGA3' R 5'GAGCTTGACAAAGTGGTCGTTGAG3'	53°C	237	MOUROT et al. [33]

Primer sequences and PCR conditions used for relative gene expression analysis.

Expressions of the *GAPDH *and β-*ACTIN *genes were used as endogenous references and oocytes from Controls were used as calibrators. Calculations of relative quantification were performed with the REST 2008 software version 2.0.7. Values are shown as n-fold difference relative to the calibrator. The evaluated transcripts were related with stress (*HSP70*), apoptosis (*BAX*), oocyte quality (*GDF9*) and oocyte developmental competence (*MATER *and *ZAR1*).

### Immunocytochemistry

Zona-intact blastocysts were fixed in 4% formaldehyde (F-1635) in PBS for 20 min, rinsed in PBS with 0.4% BSA (A-7906) for 20 min and permeabilized in 0.1% Triton-X (T-9284) in PBS for 15 min. Non-specific immunoreactions were blocked by incubation, for 30 min, in blocking solution consisting of 3% FBS and 0.2% Tween^® ^20 (H5152, Promega, Madison, WI, USA) in PBS. Incubation with the primary antibody against Oct-4(goat polyclonal IgG, SC-8628 Santa Cruz Biotechnology, Santa Cruz, CA, USA) diluted 1:100 in blocking solution, was performed for 1 h. Embryos were then rinsed in blocking solution for 15 min. Incubation with the secondary antibody (Alexa 488-donkey anti-goat IgG, A11055, Molecular Probes Inc. Eugene, OR, USA) diluted 1:1000 in blocking solution, was performed for 45 min in the dark. Embryos were then rinsed in blocking solution and counterstained with 0.01 mg/ml propidium iodide (P-4864) in blocking solution for 10 min in the dark. Blastocysts not exposed to primary antibody were used as negative controls. All the stages were performed at room temperature. Embryos were mounted in a drop of cool 70% glycerol (G-9012) in PBS on a microscope slide and covered with a coverslip, using small droplets of wax to protect embryos from collapse. Images of serial optical sections were recorded every 5μm with a confocal microscope (Olympus FV300), using software (Olympus Fluoview version 3.3) associated with a fluorescence microscope (Olympus BX 61) and 488 nm and 543 nm lasers to visualize Oct-4 positive cells and total nuclei respectively.

### Statistical analysis

Data relative to nuclear stages were analyzed by the Fisher test. Cleavage, blastocyst and hatching rates were analyzed by Chi-square test. Blastocyst total cell numbers were analyzed by one-way ANOVA, using the Graph Pad Prism 4 software. Embryo cell number on day 2 was analyzed with a three by three table and Chi-square test of independence. The proportion of Oct-4 positive cells over total cell number was analyzed by the 'Difference of proportions test' using statistical INFOSTAT (2007) software. In all cases p < 0.05 was considered to be significant.

## Results

### BMP4 and Noggin during maturation

#### Nuclear maturation and gene expression

The addition of BMP4 or Noggin to *in vitro *maturation (IVM) medium did not affect nuclear maturation under our conditions (Table 2) with most oocytes in all three groups reaching metaphase II (MII) and extruding the first polar body.

**Table 2:** Meiotic stages of oocytes

Treatment	Total	MII	MI	Other	Dead
Control	102	81 (79.4)	14 (13.7)	4 (3.9)	2(1.9)
BMP4	98	71 (72.4)	16 (16.3)	0	6 (6.2)
Noggin	89	72 (80.8)	10 (11.2)	2 (2.2)	5 (5.6)

Meiotic stages, determined by nuclear staining of oocytes matured for 24 h in the presence of 100 ng/ml of BMP4 or Noggin. Control group was matured without any supplementation (Fisher test). MII: metaphase II; MI: metaphase I. Other: anaphase and telophase

**Figure 1:**
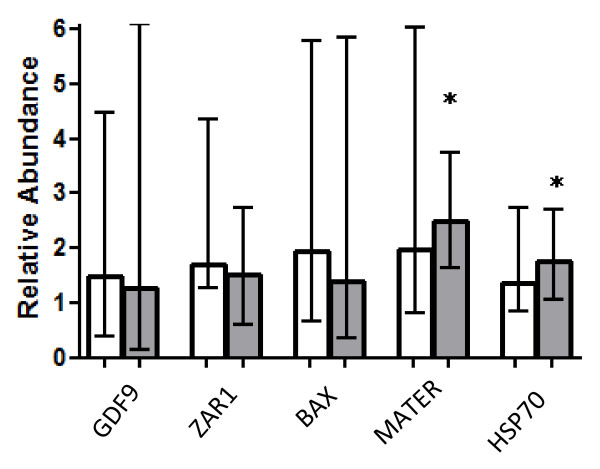
**Gene expression in treated oocytes**. Quantification of transcripts by RT-qPCR in BMP4 (white columns) and Noggin (grey columns)-treated oocytes relative to Control oocytes (mean ± extreme ratios). Statistical differences respect to Control are indicated by *(REST software). *MATER*(mean factor: 2.484) and *HSP70*(mean factor: 1.755).

No differences (p > 0.05) in the relative amount of *ZAR1, GDF9, BAX, MATER *and *HSP70 *transcripts were observed between mBMP4 and mControl groups, indicating no differential degradation or *de novo *synthesis of these messengers in oocytes matured with BMP4. On the other hand, an up regulation of *MATER *(mean factor: 2.484) and *HSP70 *(mean factor: 1.755) was observed in mNoggin oocytes (Figure 1).

#### Parthenogenic activation

After parthenogenic activation, mBMP4 and mNoggin embryos had higher (p < 0.05) cleavage rates than mControl but no effect on blastocyst production was found (Table 3).

**Table 3:** Effect of BMP4 and Noggin added to *in vitro *maturation medium on development of parthenogenic and *in vitro *fertilized embryos

	Treatment	Total	Cleaved (%)	Blastocysts (%)	Hatching blast. (%)	Blastocyst cell number ± sd
	Control	344	190 (55.2)^b^	30 (8.7)		100 ± 33
**PA**	BMP4	274	180 (65.7)^a^	22 (8.0)	1(0.4)	88 ± 14
	Noggin	242	158 (65.3)^a^	22 (9.0)		68 ± 8
	Control	249	176 (70.7)^α^	35 (14.0)	5 (2.0)	90 ± 25
**IVF**	BMP4	221	160 (72.4)^α^	28 (12.6)	4 (1.8)	120 ± 25
	Noggin	234	144 (61.5)^β^	31(13.2)	2 (0.8)	99 ± 8

Development of parthenogenic (PA) and *in vitro *fertilized (IVF) embryos produced from oocytes that were matured in the presence of 100 ng/ml of BMP4 or Noggin. Controls were matured in standard medium without supplementation. Different superscripts in PA or IVF indicate statistical differences (Chi-square test, p < 0.05). For blastocyst cell numbers, one-way ANOVA was applied

#### *In vitro *fertilization

After *in vitro *fertilization, mNoggin cleavage rate was lower (p < 0.05) than mBMP4 or mControl embryos. However, blastocyst rates were not affected (p > 0.05) indicating no effects of the treatments on advanced embryos. Similarly, the quantity of hatching embryos was not different (p > 0.05) among groups (Table 3).

No differences in embryonic cell numbers were found on day 2 (data not shown) in all groups and blastocysts from the three groups had similar total cell numbers for PA and IVF (Table 3). These data suggest that the early kinetics of development of these embryos were independent of BMP4 and Noggin during *in vitro *maturation.

#### Oct-4 expression in PA and IVF embryos

A higher proportion of Oct-4-expressing cells over total cells, was observed in mBMP4-PA blastocysts compared with mControl (p < 0.01) and mNoggin (p < 0.05) PA blastocysts. No differences among groups were found for IVF embryos (Table 4).

**Table 4:** Oct-4 expression in PA and IVF blastocysts from *in vitro *maturation experiments

	Treatment	n	Total cells	Oct-4 positive cells	% Oct-4/Total cells
**PA**	Control	3	194	122	63^b^**
	BMP4	2	100	79	79^a^
	Noggin	3	240	157	65^b^*
**IVF**	Control	2	136	103	76
	BMP4	3	151	119	79
	Noggin	3	307	218	71

Oct-4 expression in PA and IVF blastocysts produced from oocytes that were matured with 100 ng/ml of either BMP4 or Noggin, Controls were matured without supplementation. Different superscripts indicate statistical differences, 'Difference of proportions test' *p < 0.05; **p < 0.01

### BMP4 and Noggin during culture

#### Parthenogenic activation

In PA embryos, the cleavage rate and blastocyst formation were reduced (p < 0.05) by Noggin when compared to cBMP4 and cControl embryos (Table 5), showing the importance of the BMP system for early stages of development.

**Table 5:** Effect of BMP4 and Noggin added to culture medium on development of parthenogenic and *in vitro *fertilized embryos

	Treatment	Total	Cleaved (%)	Blastocysts (%)	Hatching blast. (%)	Blastocyst cell number ± sd
	Control	354	237 (66.9)^a^	48 (13.5)^a^		68 ± 33
**PA**	BMP4	295	199 (67.4)^a^	44 (14.9)^a^	1 (0.3)	91 ± 43
	Noggin	269	154 (57.2)^b^	19 (7.0)^b^		71 ± 16
	Control	218	138 (63.3)^α^	45(20.6)^α^	10 (4.6)^α^	130 ± 47
**IVF**	BMP4	217	146 (61.3)^αβ^	22 (9.2)^β^	3 (1.4)^β^	117 ± 52
	Noggin	205	105 (51.2)^β^	24 (11.7)^β^	1(0.5)^β^	128 ± 21

Development of parthenogenic (PA) and *in vitro *fertilized (IVF) embryos cultured with 100 ng/ml of either BMP4 or Noggin, Controls were cultured without supplementation. Different superscripts in PA or IVF indicate statistical differences Chi-square test (p < 0.05). For blastocyst cell numbers, one-way ANOVA was applied.

On day two of culture there was a marked tendency (p = 0.057) for cNoggin to have a higher proportion of two-cell embryos (38% vs. 29%) and a lower proportion with five to eight cells (15.6% vs. 17.7%) compared with Controls. This indicates some development retardation respect to Control. An opposite trend was observed in cBMP4, which had a higher (p = 0.057) proportion of embryos with five to eight cells (25.4% vs 17.7%) compared to Controls. No difference in blastocyst total cell number was found between groups (Table 5).

#### *In vitro *fertilization

Noggin during culture negatively affected (p < 0.05) cleavage, blastocyst and hatching rates while BMP4 addition reduced (p < 0.05) blastocyst and hatching rates of IVF embryos (Table 5).

No differences in cell number of IVF embryos on day two of culture were seen so neither acceleration nor retardation in development was observed. Similarly to PA embryos, no differences (p > 0.05) in total cell number of IVF blastocysts were found among groups (Table 5).

#### Oct-4 expression in PA and IVF embryos

**Figure 2:**
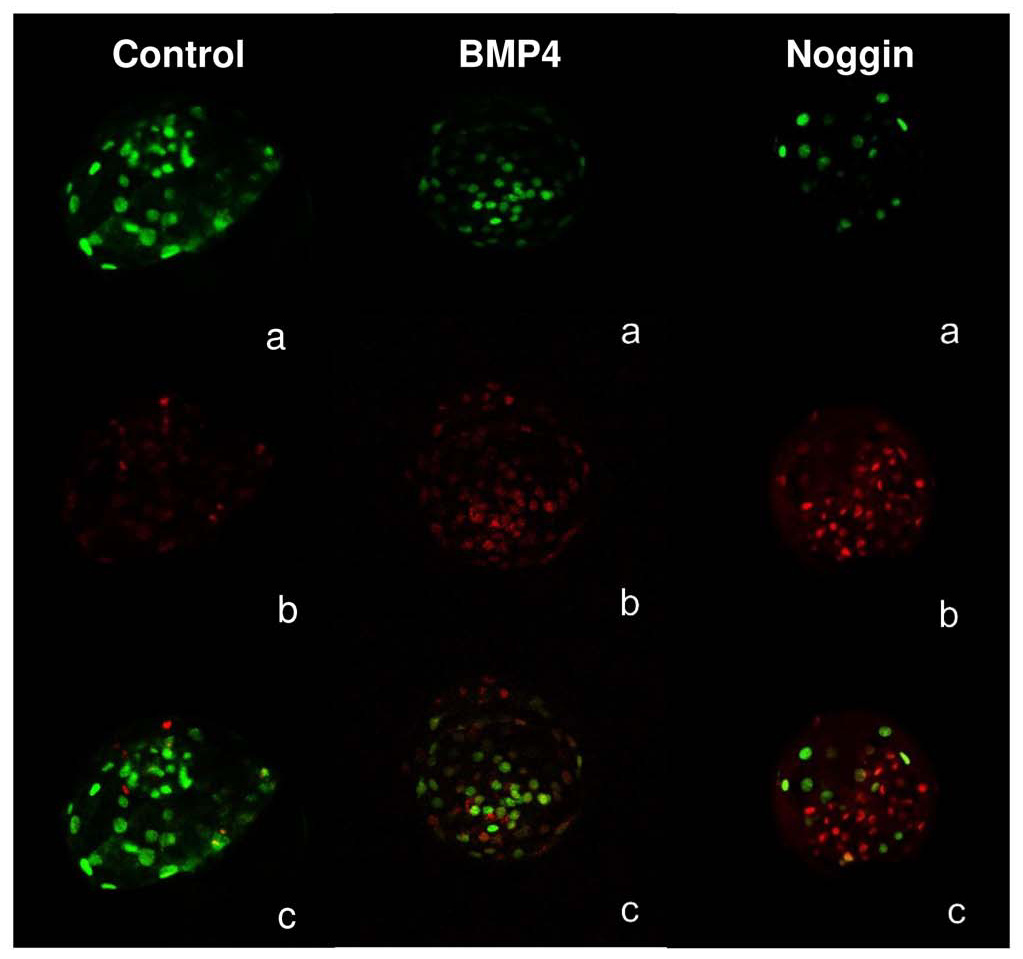
**Oct-4 expression in bovine blastocysts**. Oct-4 immunostaining of blastocysts cultured with 100 ng/ml of either BMP4 or Noggin, Controls were cultured without supplementation. Confocal microscopy, augmentation: 20X and zoom: 2. a) Oct-4 positive cells (green); b) Total nuclei (red); c) Oct-4 positive cells and total nuclei (merged).

Proportions of Oct-4-expressing cells in IVF embryos were lower (p < 0.01) in cBMP4 and cNoggin groups than in cControls (Table 6, Figure 2). No differences were found in this proportion between treatments in PA embryos (Table 6). The protein was localized in the ICM as well as in the trophoblast for all blastocysts.

**Table 6:** Oct-4 expression in PA and IVF blastocysts from *in vitro *culture experiments

	Treatment	N	Total cells	Oct-4 positive cells	% Oct-4/Total cells
**PA**	Control	3	163	129	79
	BMP4	3	180	142	79
	Noggin	2	115	84	73
**IVF**	Control	3	275	229	83^a^
	BMP4	3	324	235	72^b^**
	Noggin	3	256	185	72^b^**

Oct-4 expression in PA and IVF blastocysts. Embryos were cultured with 100 ng/ml of either BMP4 or Noggin, Controls were cultured without supplementation. Different superscripts indicate statistical differences, 'Difference of proportions test' **p < 0.01.

## Discussion

The effects of exogenous BMP4 or its inhibitor, Noggin, were studied during oocyte *in vitro *maturation or embryo culture. We found that whereas BMP4 and Noggin did not affect nuclear maturation, Noggin supplementation up-regulated the expression of *HSP70 *and *MATER *genes.

The *HSP70 *gene encodes a heat shock protein that binds several proteins and its over-expression is usually associated with stress [25]. In vascular tissues, Hsp70 protein can enhance BMP activity by binding BMP inhibitors and it can be a possible link between cellular stress and BMP signaling [26]. From our finding, we speculate that the increase of *HSP70 *transcripts could be an effort by the cell to keep BMPs at adequate levels or to minimize possible stresses induced by Noggin.

Mater is a maternal effect protein that plays an essential role on early embryo development in the mouse [27], but its role in other species is not well known. Mota et al [28] reported no variation in *MATER *gene expression between bovine oocytes with low and high competence. Whereas Pennetier et al [29] found that *MATER *mRNA amount decreases strongly during maturation and Wood et al. [30] found over-expression in oocytes from women with polycystic ovarian syndrome. Our work found a higher relative abundance of *MATER *transcripts in oocytes matured with Noggin. This could be associated with a deficient cytoplasmic maturation nevertheless we did not find differences in blastocyst rates.

We found that oocyte nuclear progression to the MII stage was not affected by the addition of BMP4 to the IVM medium which is consistent with Fatehi et al [4] results. In this work, we have also tested the effects of the inhibitor Noggin but no differences in nuclear maturation were observed.

Supplementation of media with BMP4 or its inhibitor during *in vitro *maturation had different effects on early embryonic development depending on whether the embryos were parthenogenic or *in vitro *fertilized with no effect on subsequent development. Similar results were published by Fatehi et al [4] only for maturation with BMP4 prior to IVF. However, despite blastocysts rates were similar, a higher proportion of Oct-4-expressing cells was observed in parthenogenic blastocysts obtained from oocytes matured with exogenous BMP4.

Although BMP4 has been well studied during organogenesis and extra-embryonic differentiation, BMP4 function during *in vitro *embryo development to the blastocyst stage has not been previously studied in the bovine. In a second set of experiments, we have addressed this study and BMP4 and Noggin were included in the embryo culture medium. We found that Noggin decreased cleavage, blastocyst and hatching rates of both PA and IVF embryos, showing the importance of BMP system for embryo development. This negative effect could be related to the lower proportion of Oct-4-expressing cells observed in IVF embryos. Coincidently, the same effects on blastocyst formation and Oct-4 expression pattern were observed for BMP4 during the culture of IVF embryos. In mice, Murohashi et al [31] observed a lower ratio of ICM-derived cells over trophoectoderm-derived cells in blastocysts cultured and treated with Noggin, than Controls. It is possible that our results were related to similar effects on the pluripotency capacity of the embryos.

Although BMP4 addition to the culture medium did not affect cleavage rates for PA or IVF embryos, the rate of blastocyst production was reduced for IVF embryos, but not for PA embryos. These results suggest differences, in the BMP system, between PA and IVF embryos. This could be caused by the different imprinting pattern of both types of embryos [32]. PA embryos only contain maternal genome which could, in turn, cause differences at many levels e.g. expression of BMP ligands, receptors, intracellular mediators, etc.

In summary, we proof that Noggin, as BMP4, did not affect oocyte nuclear maturation. Noggin supplementation up-regulated the expression of *HSP70 *and *MATER *genes in matured oocytes. Moreover, BMP4 during maturation increased the proportion of Oct-4 positive cells in parthenogenic embryos. On the other hand, when Noggin was added to embryo culture medium, developmental rates of parthenogenic and *in vitro *fertilized embryos were reduced. However, BMP4 addition decreases the development only for *in vitro *fertilized embryos. BMP4 and Noggin during culture reduced the proportion of Oct-4-expressing cells.

## Conclusions

Our results show that BMP4 is implicated in bovine oocytes maturation with effects on cleavage rates and pluripotent state of blastocyst cells; and Noggin modifies the expression of some genes in oocytes. Moreover our findings demonstrate, for the first time, that a correct balance of BMP signaling is needed for proper pre-implantation development of bovine embryos.

## Competing interests

The authors declare that they have no competing interests.

## Authors' contributions

ILR performed all the experiments but the RT-qPCR measurements and drafted the manuscript. LSAC contributed to the RT-qPCR analysis and gave critical discussion to the work. MMP performed the RT-qPCR experiments. RFM contributed in experiment design and draft of the manuscript. DAP contributed in experiment design and with immunocytochemistry experiments and DFS contributed in experiment design and draft of the manuscript. All authors read and approved the final manuscript.
